# Lead-to-Port Reversal Mimicking Loss of Cardiac Resynchronization Therapy After CRT-D Generator Replacement

**DOI:** 10.1016/j.jaccas.2026.107717

**Published:** 2026-03-28

**Authors:** Ahmet Taha Sahin, Serhat Kesriklioglu, Ahmet Lutfu Sertdemir, Enes Elvin Gul

**Affiliations:** Department of Cardiology, Necmettin Erbakan University, School of Medicine, Konya, Turkiye

**Keywords:** cardiac resynchronization therapy, device interrogation, electrocardiogram, left ventricle, pacing troubleshooting

## Abstract

**Background:**

Lead-to-port misconnections are a rare but clinically significant complication after cardiac device generator replacement and may mimic true lead failure or loss of capture, leading to diagnostic confusion and unnecessary interventions.

**Case Summary:**

A 72-year-old man with ischemic cardiomyopathy and a previously implanted cardiac resynchronization therapy defibrillator presented with worsening dyspnea following elective generator replacement performed at another center. Postprocedural electrocardiogram demonstrated loss of biventricular pacing. Chest radiography confirmed correct lead positions. High-output left ventricle (LV) pacing failed to produce capture, while unipolar LV pacing resulted in surface P waves, and AAI pacing revealed ventricular capture. Stepwise device interrogation excluded lead dislodgement and malfunction, establishing inadvertent LV–right atrium port reversal during generator replacement as the underlying cause.

**Discussion:**

This case highlights how systematic pacing maneuvers and careful electrocardiogram interpretation can rapidly differentiate electrical misconnection from mechanical lead failure, preventing misdiagnosis and inappropriate lead revision.

## Graphical Abstract

**Figure:**
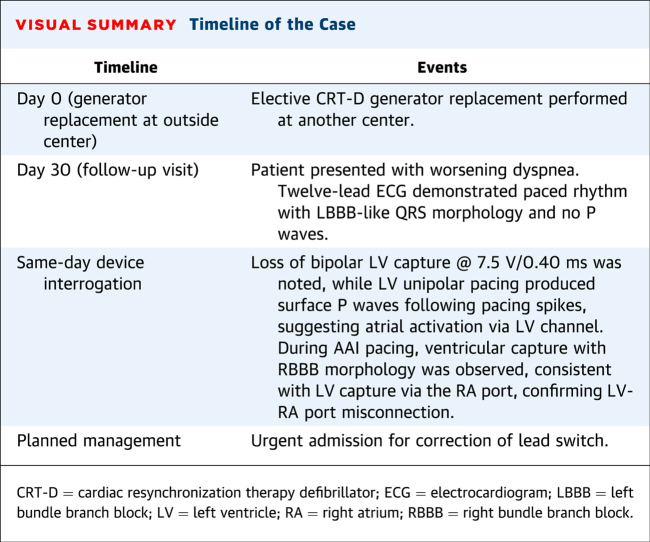

## History of Presentation

**Figure 1:**
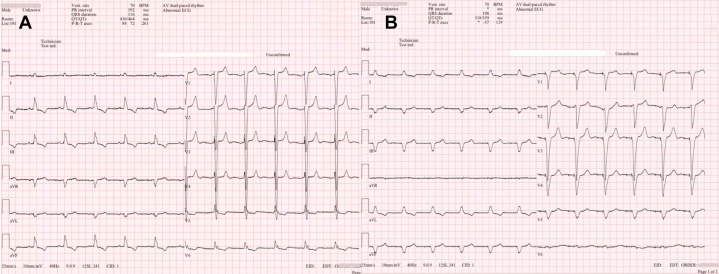
Baseline and Post–Generator Replacement Electrocardiograms (A) Prereplacement 12-lead ECG showing biventricular pacing with LBBB morphology. (B) One-month follow-up after generator replacement, 12-lead ECG demonstrating RV-paced morphology with negative concordance in precordial lead and superior axis. ECG = electrocardiogram; LBBB = left bundle branch block; RV = right ventricle.

A 72-year-old man with ischemic cardiomyopathy and a left ventricular ejection fraction of 25% presented to our clinic 1 month after elective cardiac resynchronization therapy defibrillator (CRT-D) generator replacement performed at another center. He reported worsening dyspnea since the replacement procedure. Prior to generator change, 12-lead electrocardiogram (ECG) demonstrated biventricular pacing with left bundle branch block morphology (Figure 1A). At the 1-month visit, ECG revealed paced rhythm with loss of typical biventricular morphology (Figure 1B). Device was programmed DDD at 60 beats/min with sensed and paced atrioventricular (AV) delays of 130 and 150 ms, respectively, and left ventricle (LV) offset programmed 20 ms ahead of right ventricle.

**Take-Home Messages**

• Lead-to-port misconnections are a rare but completely preventable cause of apparent pacing failure after device replacement.
• Recognition of paradoxical electrocardiogram findings should prompt immediate evaluation for header reversal.
• Careful verification of lead connections along with a 12-lead electrocardiogram assessment before pocket closure is essential to avoid unnecessary interventions and ensure safe device function.

## Past Medical History

The patient had ischemic cardiomyopathy with reduced ejection fraction, treated previously with CRT-D implantation using a bipolar LV lead. No prior lead revisions or sensing/capture problems were documented before generator exchange.

## Differential Diagnosis

Based on new ECG morphology and clinical deterioration, the leading diagnostic possibilities included:

• LV lead failure or loss of capture
• LV lead dislodgement
• LV–right atrium (RA) lead/port misconnection during generator replacement
• Atrial sensing abnormalities or atrial undersensing

Given the preserved lead position on imaging and paradoxical ECG findings, mechanical dislodgement alone was considered less likely.

## Investigations

**Figure 2:**
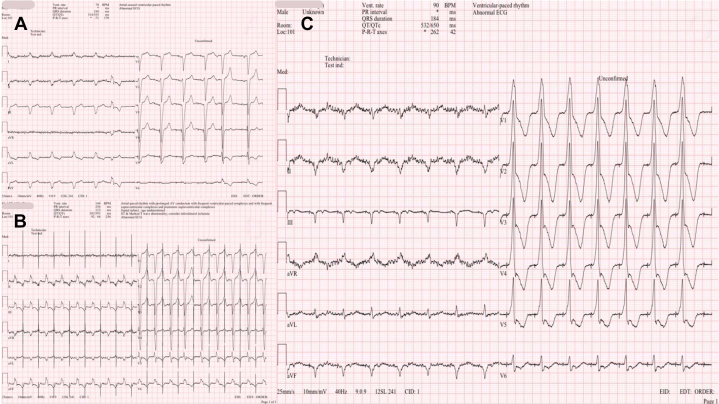
Stepwise Diagnostic Maneuvers (A) No LV capture at high output (LV 1-2 @ 7.5 V/0.40 ms). (B) Unipolar LV pacing produces surface P waves following the unipolar pacing spikes. (C) AAI pacing shows RBBB morphology compatible with LV-only pacing. LV = left ventricle; RBBB = right bundle branch block.

**Figure 3:**
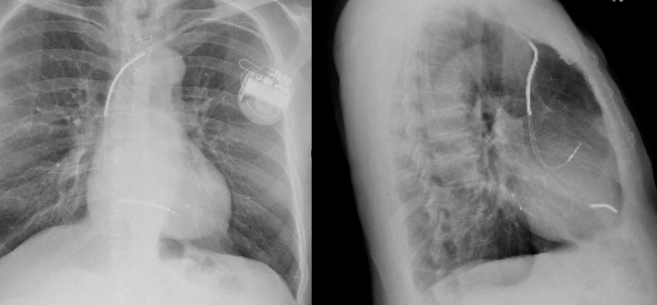
2-View Chest X-Ray Reveals all 3 Leads in Place Including the Bipolar Left Ventricular Lead, Which Is the Posterolateral Branch

Stepwise device troubleshooting was performed. Incremental bipolar LV output testing up to 7.5 V/0.4 ms failed to achieve LV capture (Figure 2A). When the LV channel was switched to unipolar pacing (LV1–can), surface P waves appeared immediately after pacing spikes, indicating atrial myocardial activation from the LV channel (Figure 2B). Conversely, AAI pacing generated a paced QRS with right bundle branch block morphology, demonstrating true LV capture via a channel not labeled as LV (Figure 2C). Two-view chest radiography confirmed that all leads, including the bipolar LV lead, were anatomically positioned correctly in the posterolateral coronary sinus branch (Figure 3). These findings collectively indicated that the atrial lead was functionally connected to the LV channel, consistent with inadvertent LV–RA port reversal during generator replacement.

## Management

**Figure 4:**
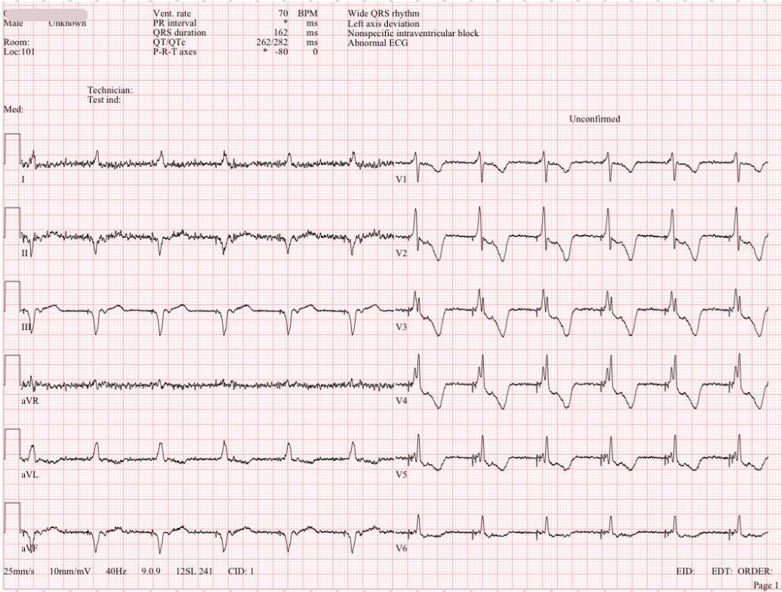
Final 12-Lead ECG in DDDR @ 70 beats/min, With Shortest AV Delay of 15 ms and RV-Only Pacing AV = atrioventricular; DDDR = dual-chamber rate-responsive pacing mode; ECG = electrocardiogram; RV = right ventricle.

Following confirmation of LV–RA misconnection, interim device programming adjustments were implemented to ensure hemodynamic stability and safety. SmartMode was disabled to avoid inappropriate dual-chamber discrimination, LV sensing was turned off, the device was programmed to the dual-chamber rate-responsive pacing mode with the shortest AV delay, and pacing was temporarily limited to the right ventricle channel (Figure 4). Urgent correction of lead-to-port header connection and re-establishment of effective biventricular pacing were planned.

## Outcome and Follow-Up

The patient was scheduled for urgent revision to correct the LV–RA reversal. Restoration of appropriate lead configuration and reprogramming were planned to re-establish biventricular resynchronization and improve clinical symptoms.

## Discussion

Lead-to-port reversal represents an uncommon but clinically relevant complication following generator replacement procedures and may closely mimic true lead malfunction or loss of capture, leading to diagnostic uncertainty and inappropriate interventions. Similar header misconnection events have been reported in both dual-chamber pacing systems and CRT devices, where the paradoxical pacing behavior rather than lead displacement or structural failure accounted for the observed abnormalities.

Naegeli and Bertel^1^ first demonstrated that atrial–ventricular header misconnection could produce apparent crosstalk phenomena and double pacing spikes despite anatomically well-positioned leads, highlighting how purely electrical misassignment can masquerade as device failure. Viswanathan et al^2^ subsequently described reverse pacemaker-mediated tachycardia due to lead reversal, where the device alternately stimulated and tracked atrial and ventricular events through incorrectly assigned channels. Jastrzebski^3^ further emphasized that the same technical error may manifest either as tachycardia or asystole depending on the interplay between intrinsic AV conduction and programming characteristics, underscoring the wide electrophysiologic heterogeneity of this complication. Additional reports have further expanded understanding of this complication. Hamdi and colleagues described a pacemaker-dependent patient in whom a header reversal remained silent for years until atrial fibrillation induced ventricular inhibition due to far-field oversensing.^4^ Ozeke et al^5^ illustrated a pin-port misconnection leading to endless-loop tachycardia following generator replacement, underscoring how even routine procedures can precipitate complex pacing malfunctions. Most recently, Katheria et al. proposed a structured diagnostic algorithm based on sequential single-chamber pacing maneuvers—switching between AAI and VVI modes—to confirm channel identity when ECG and electrogram data appear incongruent.^6^ These landmark cases highlight how lead misconnection can present with wide morphological variability, from apparent capture loss to reentrant tachyarrhythmia and even to more life-threatening conditions like syncope. This simple bedside strategy remains invaluable for rapidly differentiating electrical malfunction from mechanical or programming errors.

**Figure 5:**
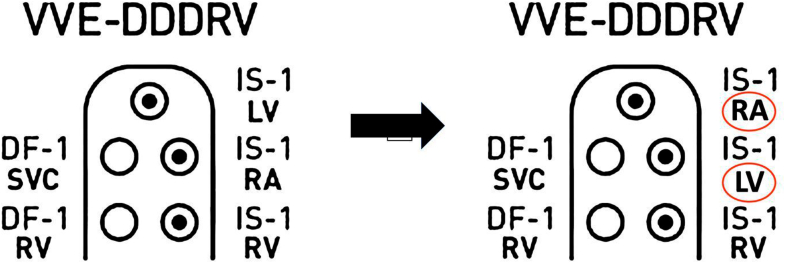
The Illustration of DF-1/IS-1 CRT-D With Lead-to-Port Misconnection of Bipolar LV–RA Leads CRT-D = cardiac resynchronization therapy device; LV = left ventricle; RA = right arterial.

Our case contributes to this growing body of evidence by illustrating LV–RA port reversal in a CRT-D system, resulting in the functional loss of biventricular pacing and symptom deterioration despite anatomically intact leads (Figure 5). Recognition of unipolar pacing–induced surface P waves and discordant atrial electrogram timing played a pivotal diagnostic role. Sequential pacing maneuvers allowed rapid bedside differentiation of electrical misconnection from mechanical lead failure, avoiding unnecessary invasive intervention. Following device interrogation, temporary programming adjustments were implemented to ensure hemodynamic stability while definitive correction was arranged.

## Conclusions

This case highlights LV–RA port reversal as a rare but entirely preventable cause of apparent pacing failure after CRT-D generator replacement. Systematic pacing maneuvers and careful ECG interpretation allow accurate diagnosis and prevent unnecessary lead revision.

## Funding Support and Author Disclosures

The authors have reported that they have no relationships relevant to the contents of this paper to disclose.

**Visual Summary:** Timeline of the Case

Timeline	Events
Day 0 (generator replacement at outside center)	Elective CRT-D generator replacement performed at another center.
Day 30 (follow-up visit)	Patient presented with worsening dyspnea. Twelve-lead ECG demonstrated paced rhythm with LBBB-like QRS morphology and no P waves.
Same-day device interrogation	Loss of bipolar LV capture @ 7.5 V/0.40 ms was noted, while LV unipolar pacing produced surface P waves following pacing spikes, suggesting atrial activation via LV channel. During AAI pacing, ventricular capture with RBBB morphology was observed, consistent with LV capture via the RA port, confirming LV-RA port misconnection.
Planned management	Urgent admission for correction of lead switch.

CRT-D = cardiac resynchronization therapy defibrillator; ECG = electrocardiogram; LBBB = left bundle branch block; LV = left ventricle; RA = right atrium; RBBB = right bundle branch block.
